# The Use of Superb Microvascular Imaging in Evaluating Rheumatic Diseases: A Systematic Review

**DOI:** 10.3390/medicina59091641

**Published:** 2023-09-11

**Authors:** Goda Seskute, Gabija Jasionyte, Rita Rugiene, Irena Butrimiene

**Affiliations:** 1 Clinic of Rheumatology, Orthopaedics Traumatology, and Reconstructive Surgery, Institute of Clinical Medicine, Faculty of Medicine, Vilnius University, LT-01513 Vilnius, Lithuania; gabija.jasionyte@santa.lt (G.J.); rita.rugiene@santa.lt (R.R.); irena.butrimiene@santa.lt (I.B.); 2 Department of Experimental, Preventive and Clinical Medicine, State Research Institute Centre for Innovative Medicine, LT-08406 Vilnius, Lithuania

**Keywords:** superb microvascular imaging, microflow imaging, microvascular imaging, low flow, autoimmune rheumatic diseases, ultrasonography, systematic review

## Abstract

*Background and Objectives*: Superb microvascular imaging is an advanced Doppler algorithm that seems to be useful in detecting low-velocity blood flow without using a contrast agent. Increasing evidence suggests that SMI is a more sensitive tool than conventional Doppler techniques for evaluating rheumatic diseases, especially inflammatory arthritis. We aimed to assess the use of SMI in evaluating joints and extraarticular structures. *Materials and Methods*: Two reviewers independently reviewed the literature to provide a global overview of the possibilities of SMI in rheumatology. Original English-language articles published between February 2014 and November 2022 were identified through database (PubMed, Medline, Ebsco, the Cochrane Library, and ScienceDirect) searching, and analysed to summarise existing evidence according to PRISMA methodology. Inclusion criteria covered original research articles reporting applications of SMI on rheumatic diseases and musculoskeletal disorders secondary to rheumatic conditions. Qualitative data synthesis was performed. *Results*: A total of 18 articles were included. No systematic reviews fulfilled our inclusion criteria. Most studies focused on characterising the synovial vascularity of rheumatoid arthritis. There have been several attempts to demonstrate SMI’s value for evaluating extra-articular soft tissues (fat pads or salivary glands) and large-diameter vessels. The quantitative importance of SMI vascular indices could become a useful non-invasive diagnostic marker. Studies on therapeutic applications are still scarce, and the majority of studies have gaps in reporting the methodology (ultrasound performance technique and settings) of the research. *Conclusions*: SMI has proved to be useful in characterising low-flow vascularity, and growing evidence indicates that SMI is a non-invasive and lower-cost tool for prognostic assessment, especially in inflammatory arthritis. Preliminary findings also suggest potential interest in evaluating the effect of treatment.

## 1. Introduction

In the last decade, ultrasound (US) has become a widely used imaging tool in rheumatology. It has many advantages, including real-time and cross-sectional imaging with excellent spatial resolution. Conventional power Doppler (PD) imaging is accepted as the non-invasive ‘gold standard’ in the assessment of vascularity in active synovitis [1,2], but it is not very sensitive to low-flow microvascular patterns [3]. Synovial proliferation, which cannot be delineated by PD, may be demonstrated clearly using contrast-enhanced ultrasound (CEUS). It is a well-established imaging modality that provides real-time visualisation of contrast enhancement patterns in various organs. As has been proved by most studies in recent years, CEUS can indicate early arthritis with a high sensitivity to microvascularisation and is even consistent with histopathological changes in inflammatory arthritis [4,5]. However, the lack of availability, the high price of the intravenous contrast agent, the unclear standard of normal articular perfusion patterns, and the challenge to repeat the examination are the main limitations of the daily clinical application of CEUS in the evaluation of joint inflammation [6]. 

New advances in microvascular imaging have been developed. In 2014, Hata [7] and Lim [8] first announced the advanced Doppler technique presented by Toshiba Medical Systems: superb microvascular imaging—SMI (Canon Medical Systems Corporation, Tokyo, Japan). Its novel algorithm differentiates low-velocity blood-flow signals from clutter and motion artefacts and enables the detection of microvessels without the need for contrast enhancement. The clinical application of SMI has become a new target for researchers. Increasing evidence suggests that SMI detects synovial (even subclinical) vascularity more sensitively than conventional PD and identifies the real remission of arthritis. SMI is proven to be useful in different fields such as oncology, cardiology, rheumatology, gastroenterology, and even dermatology [7,8,9]. 

The aim of this study was to systematically review the literature on a global overview of the use of SMI for assessing rheumatic diseases. The results will inform clinicians on evidence-based vascularity detection using SMI in patients with arthritis and other rheumatic diseases. The review will also identify research gaps. 

## 2. Materials and Methods

We adopted the guidelines of the Preferred Reporting Items for Systematic Reviews and Meta-Analyses (PRISMA) statement for conducting the systematic review and reporting its results [10].

### 2.1. Literature Search Strategy

PubMed, Medline, Ebsco, the Cochrane Library, and ScienceDirect were searched without any date restriction, starting from the first articles on the application of SMI in February 2014 [7,10]. The MeSH terms were used in our search strategy: ‘ultrasonography, Doppler’. The following keywords were used: ‘superb microvascular imaging’, ‘microvascular imaging’, ‘microflow imaging’, ‘low flow’. We also checked the references of all retrieved articles for other relevant publications. 

### 2.2. Eligibility Criteria

We included studies that were (1) original research articles reporting rheumatological applications of SMI (regardless of whether they were compared with other imaging modalities); (2) reporting musculoskeletal disorders evaluated by SMI which are secondary due to rheumatic conditions; (3) written in English; (4) full text, and in which the complete data were available. Narrative reviews, pictorial essays, case reports, and case series were excluded from our analysis.

### 2.3. Study Selection and Analysis

Two reviewers independently performed the literature search and screened titles with abstracts and full texts according to the inclusion criteria. Any disagreements among reviewers were discussed until a consensus was reached. The following data were collected from each paper included in the final analysis: the authors, country, year of publication, rheumatic disease, sample size, scanned joints, details about US technique (used probes, scanned planes, and modes of SMI), SMI comparison tools and scoring system. Qualitative data synthesis was performed and results were tabulated for visual comparison summarising characteristics of the presented studies (Supplemental Table S1).

## 3. Results

**Figure 1:**
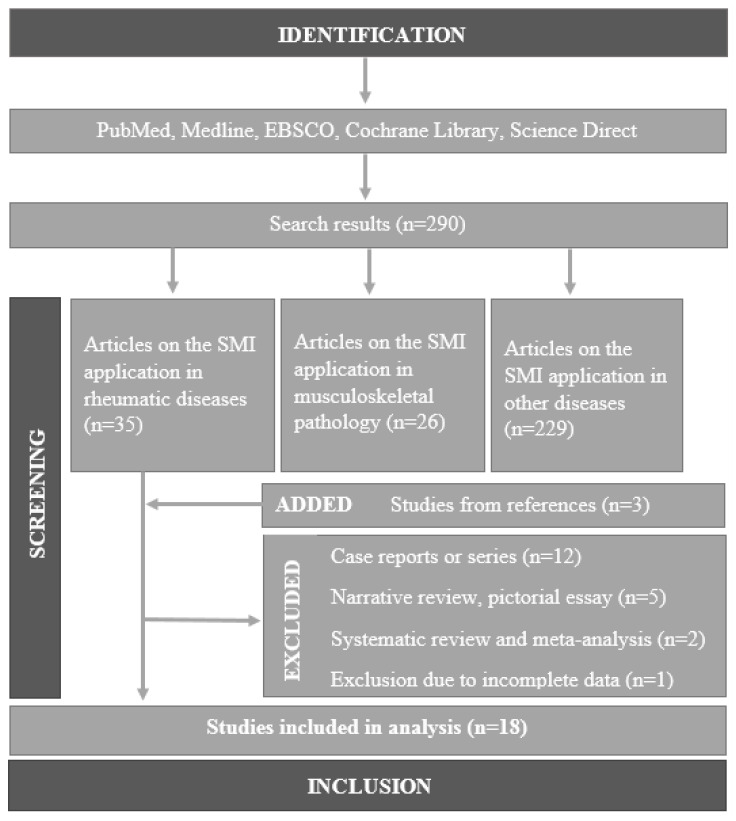
A flow diagram depicting the literature search and study selection.

A total of 290 articles were identified after the initial search. After reviewing the titles and abstracts, we obtained 35 articles and three relevant publications were found in the references of all of the retrieved articles. Hence, 18 studies were included in the systemic review based on the inclusion criteria. A schematic illustration of the literature screening and selection process is presented in the flow chart (Figure 1). We did not identify any systematic reviews fulfilling our inclusion criteria. One systematic review was found analysing overall musculoskeletal applications of SMI, and one meta-analysis of the SMI scoring system in rheumatoid arthritis (RA). 

The included 18 studies were published between August 2016 and May 2022. Overall, 19 original studies of SMI application in rheumatology were found and 18 [11,12,13,14,15,16,17,18,19,20,21,22,23,24,25,26,27,28] of them were analysed because we were not able to obtain the full text of one publication. The majority of the included studies were conducted in Japan [11,12,13,14,15], China [16,17,18,19,20], and Turkey [21,22,23,24]; single studies were conducted in Australia [25], Italy [26], South Korea [27] and the United Kingdom [28]. Reference screening provided three additional papers, but only one fulfilled the inclusion criteria and was included for analysis [20].

Supplemental Table S1 depicts the baseline characteristics of the identified studies and enrolled patients. The most studied rheumatic disease was RA (52.63% of studies) [11,12,13,14,16,18,19,20,26] representing up to 63.15% of the studies when analysed in a mixed sample together with non-RA arthritis [15,28]. The main target of the research was synovial vascularity of the joints (84.21%). The assessment of active synovitis in knee osteoarthritis (OA) by using SMI and PD was performed [25], but the distinction between RA (or other inflammatory arthritis) and OA was not established. Two studies involved patients with OA in the non-RA control group [20] or as subjects with arthralgia without specific distinguishment of ultrasound findings between both diseases [28]. Juvenile idiopathic arthritis, psoriatic arthritis, and other non-RA arthritis were mixed in samples with RA patients [25,26]. In this case, the main target of evaluation was non-specific synovial vascularity. 

There were several attempts to demonstrate the usefulness of SMI for evaluating extra-articular soft tissues (fat pads [23] or salivary glands [24], and large vessels [22]). One study included arthritis-prone rats [14]. The evaluation of joints has three characteristics: one or a mixed group of joints; unilateral or bilateral; small or large joints. Wrist, knee, and hand (metacarpophalangeal—MCF, and proximal interphalangeal—PIF) joints were the most widely investigated joints. SMI was mostly compared with conventional PD [14,15,18,19,20,21,22,23,24,25,26,27,28], and several studies included colour Doppler in their comparison methods [18,24]. A semi-quantitative scale (four-point 0–3 or 0–III grades by percentage, dots, or linear signals in the part of the synovial area) was adopted widely to assess vascularity using SMI and other Doppler techniques (14 out of 18 analysed studies). There appears to be a new tendency to prefer the vascularity index as a quantitative measure [14,23,24]. The main purpose of the studies was to demonstrate the usefulness and priority of SMI in detecting synovial vascularity in different stages of the disease, and treatment options. Final remarks justified the purposes and affirmed that SMI has higher sensitivity compared with PD in the depiction of low-grade synovial vascularity. 

The greatest heterogeneity among studies was the assessed Doppler parameters, sample size, disease activity, and the vascularity scoring systems. To sum up, SMI was mostly used to detect low-grade synovial vascularity in small joints (MCF, interphalangeal) of RA for evaluating disease activity or true clinical remission. There is a lack of data about the use of SMI in assessing other arthritides (e.g., OA, gout), extraarticular rheumatic disorders (enthesitis, tenosynovitis, myositis, vasculitis, sialoadenitis), or therapeutic effects. Comparison with other modalities than PD, especially CEUS, is also a priority. 

**Figure 2:**
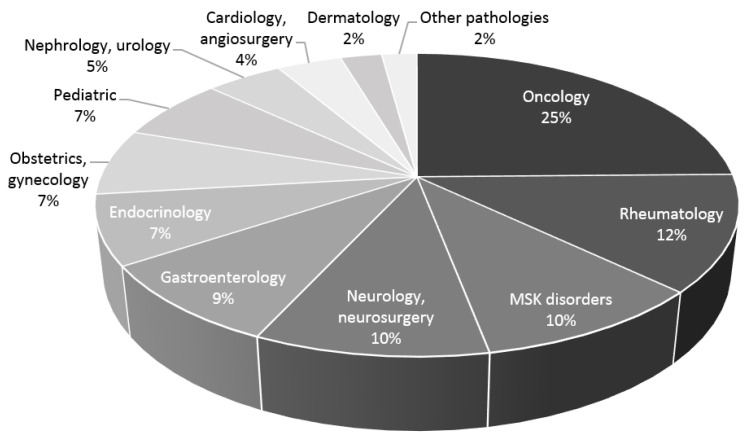
The distribution of SMI applications in different clinical fields according to the titles and abstracts of articles.

The additional analysis of all diseases according to the titles showed that the usefulness of SMI for diagnosing a wide spectrum of diseases is still growing and that the field of rheumatology takes second place, after oncology (Figure 2). 

## 4. Discussion

The first applications of SMI in rheumatology were demonstrated by case reports in 2014 [7,10], and according to the number of original studies, the peak was reached in 2020 [13,18,23,24,25]. The use of SMI in the field of rheumatology remains sluggish (only 19 original articles in almost a decade) despite ranking in the top three by field (Figure 2). There is an explanation for this statement. Ultrasound devices are able to provide diagnostic capabilities at a much lower cost than other imaging tools such as computed tomography or magnetic resonance imaging (MRI). Still, even the advanced US modalities require high financial resources and are not always prioritised as a necessity for daily patient care [29,30]. The availability of SMI in rheumatology and other fields has spread slowly due to limited access to the expensive equipment. In addition, US scans are performed mostly by radiologists; a lack of experience or opportunities for learning US imaging is also an important issue.

Sustained and clinical remission is an attainable treatment goal in the management of patients with arthritis [31]. The frequency of achieved sustained remission is increasing over time, especially due to targeted treatment strategies. Disease activity should be monitored and therapy should be adjusted during the whole disease course. The Boolean American College of Rheumatology/European League against Rheumatism activity index (ACR/EULAR) remission score includes clinical and laboratory measures [32]. According to the literature, clinical remission, even classified by strict composite indices, does not seem to reflect the “true” remission. Filippou et al. showed that 80.9% of patients with RA in remission had at least one form of synovitis or tenosynovits in B-mode, with PD in 51% of patients [33]. Ultrasonography appears to be the best way to assess real remission. According to Han et al., the risk of recurrence was 4.5 greater for those with PD positivity than for those with PD negativity [34]. Traditionally, PD is the most popular reference modality of imaging for detecting synovial vascularity in RA patients [35,36], but it is limited in the detection of microvascular patterns and low blood flow velocity. However, the majority of included studies are focused on RA and analyse subclinical synovitis in the joint using SMI rather than conventional PD. Compared to conventional PD, the advantages of SMI are high frame rates, high sensitivity in visualising vessels with low velocities, high spatial resolution, and low motion artefacts. SMI separates low-flow signals from overlaying tissue motion artifacts [7]. This allows one to detect small vessels of neoangiogenesis in the synovium never seen before with ultrasound. Analysing remission with both modalities reveals the strength of SMI to evaluate subclinical synovitis. Orlandi et al. first made a point that SMI is more sensitive than PD in RA patients in remission under treatment with rituximab [26]. SMI compared with PD improves the total rate of detection of abnormal vascularisation by 24.3% for inactive RA patients [20]. Yu et al. analysed 572 joints (52 wrist, 260 PIF, and 260 MCF) and reported that SMI could aid the identification of true remission in RA patients. The remission rate identified using PD was 65.4%, and 42.3% using SMI, in 26 RA patients in clinical remission [19]. However, the capability of SMI to detect all low-velocity microvessels is difficult due to its limited penetration. CEUS is able to visualise all small blood vessels by the contrast agent circulating through capillary beds. Contrast microbubbles generate high-intensity signals that are detected by the transducer. There is only one study about the sensitivity of SMI in detecting vascularisation of the synovial membrane in RA patients with clinical remission compared to CEUS. Diao et al. showed that the detection rates of positive synovial vascularity in clinical remission using SMI and CEUS were 79.2% (95% CI: 67.2–91.1%) and 83.3% (95% CI: 72.4–94.3%), respectively [16]. CEUS is not a routine assessment for arthritis because of the imposition of time limitations or contrast agent injection. These disadvantages establish the priority of SMI, especially for patients fearful of injections. On the other hand, both SMI and CEUS can lead to incorrect diagnosis or over-treatment because clinicians are not able to discriminate between pathological and physiological findings in the joints commonly affected by inflammatory arthritis conditions. Thus, imaging remission could be used to improve the prognosis of RA patients. More studies with large groups of healthy patients and a comparison of methods (SMI vs. CEUS) are needed. 

Enthesitis is one of the most common symptoms of spondyloarthropathies (SpA), especially ankylosing spondylitis (AS), and seems to be the earliest lesion in animal models of SpA [37]. Enthesitis has been considered to be a focal insertional disorder, but advanced imaging and pathologic findings suggest that it is a diffuse process involving surrounding bone and soft tissues [38]. Enthesitis is often underdiagnosed in clinical assessment and poorly correlated with markers of inflammation. Ultrasound is able to detect pathological changes in enthesitis at both early and late stages. Grey-scale enthesitis is characterized by thickness and echogenicity changes, the detection of enthesophytes, erosions, calcifications, associated bursitis, and cortical irregularities. The vascularity assessment is performed using Doppler modalities. There is only one study about the diagnostic value of SMI in the assessment of lateral epicondylitis (as a primary musculoskeletal disorder) [39], but SpA-induced enthesitis has still not been evaluated. The neovascularization of lateral epicondylitis was detected much better with SMI compared to colour and power Doppler modalities, and the combination of SMI and B-mode sonography was found to have excellent diagnostic performance. A large group of entheses could be involved in SpA, and the methodical approach to it could be one of research. This would have an additional role in the treatment management of SpA. Also, Ünal et al. evaluated quadriceps and patellar tendons in juvenile idiopathic arthritis with a combination of SMI and shear wave elastography (SWE) [23]. Ahn et al. investigated the clinical feasibility of ultrasound elastography for assessing patients with lateral epicondylitis (the cause is unknown) [4]. Elastography revealed a soft area on 73 of 97 tendons (75.3%), and the significantly lower mean strain ratio of the symptomatic tendons indicates that these tendons were softer than asymptomatic [40]. B-mode sonography, SMI, and SWE could be a cheap, reliable package of tests in the real-time evaluation of structural changes, neovascularity, and elasticity in enthesitis of SpA or other periarticular structures (recesses, tendons, salivary glands) damaged by rheumatic diseases. This undisclosed field is another direction for research.

Other noteworthy applications of novel US research included primary Sjögren’s syndrome (pSS), low-grade OA, and Buerger’s disease. It seems that SMI has become a multidisciplinary diagnostic approach for rheumatic diseases. Ustabaşıoğlu et al. used the vascularity index of SMI for imaging salivary glands [24]. SMI compared to PD has a higher sensitivity and specificity in diagnosing pSS, especially when used with clinical, laboratory, and other imaging methods. The sensitivity and specificity of SMI vs. PD for the diagnosis of pSS in the parotid gland were 87.5% vs. 82.5% and 72.5% vs. 70%; for the diagnosis of pSS in the submandibular gland, they were 82.5% vs. 77.5% and 70% vs. 67.5%. SMI was compared with PD and MRI in the assessment of active synovitis in knee OA, and regardless of SMI superiority, the added clinical value was still not clear [25]. SMI provided a superior demonstration of collateral vessel formation using both modes in Buerger’s disease [22]. Interestingly, the advanced microflow imaging seems to be a reliable imaging tool for assessing vessel-wall morphology and flow waveform characteristics in the case of vertebral artery dissection, atherosclerosis (plaque instability in carotid arteries), and mobile intracardiac structures [41,42]. This suggests that SMI may improve the diagnostic accuracy of detecting inflammatory vascular lesions in vasculitis. There are several case reports in the literature that have demonstrated the use of SMI in detecting aortitis caused by large-vessel vasculitis: microscopic polyangiitis [43], giant cell arteritis [44], and Takayasu arteritis [45,46]. The assessment to evaluate aortitis with SMI has been approved using CEUS and MRI or even positron emission tomography. SMI seems to be able to detect the middle layer (media) of the aortic wall within the hypoechoic, periaortic rim, consistent with neovascularization (or intramural vascularization) [39,41,42]. The most essential methodological tips for evaluating vessels are performing US images in both planes (transverse and longitudinal), using both modes, and comparing this with other radiological tests at baseline. SMI may be a potentially helpful tool in the assessment of disease activity in patients affected by large-vessel vasculitis; thus, it requires further investigation. 

The degree of synovitis was determined semi-quantitatively in 77.77% (14 of 18) of analysed studies. A heterogenous four-point visual analogue scale is used to compare the PD and SMI based on the sensitivity and resolution of the vessels visualised in the region of interest (ROI). Lin et al. compared semi-quantitative scoring systems in detecting synovitis in RA patients, and the pooled summary odds ratio was 2.12 with statistical significance, which strongly suggests that SMI modality is more sensitive than conventional PD [47]. There is a limitation in analysed studies on whether the grades of SMI and PDI interacted with blinding. On the other hand, there is currently no concept of a scoring system and vascular signals are measured in different units (in percentage of the ROI filled, volume of dots, and linear flow). These small methodical details also produce inconsistent interpretations of synovial vascularity, although Szkudlarek et al. introduced a scoring system that remains the basis for this issue [48]. The vascular index (VI) is a quantitative parameter for SMI and PD corresponding to the ratio of coloured pixels to the total pixels within the selected ROI [20]. VI can provide more objective information regarding synovial vascularity. Mean VI values are calculated by averaging the results of three different acquisitions of PD and SMI, which helps to improve diagnostic performance [49]. A proportion of 3 out of 18 analysed studies used VI for measurements [14,23,24]. Horie et al. first determined PD and SMI values as ‘the number of pixels’ in a joint space [14]. It was a little complicated to delineate the boundaries of the synovium in the small ankle joint of the rat. ROI was selected methodically but values were determined using a separate tool for analysis (‘ImageJ, 1.50i’) in manually defined ROI [14]. Ünal et al. first presented a definition of VI and a methodical technique for measuring it in joint synovium automatically using SMI and PD [23]. They depicted a region from the recess, including the synovium, because focusing on the synovium alone by outlining the synovial borders is time-consuming. The range of cut-off values for VI between 4.15% and 5.45% was defined for the diagnosis of acute arthritis [23]. Ustabaşıoğlu et al. demonstrated the positioned ROI at the centre of the gland, and automatically calculated the VI [24]. Quantitative SMI VI values could become a useful non-invasive diagnostic marker for clinicians, but more studies are needed.

SMI presents two modes: colour (cSMI, which demonstrates B-mode and colour information simultaneously) and monochrome (mSMI, which focuses only on the vasculature) [7]. Both modes demonstrate the value in differentiating a wide variety of clinical situations: benign and malignant tumours, the therapeutic effect of the treatment, inflammatory diseases, and many other medical conditions [50,51]. Only Nas et al. used and demonstrated both SMI modes’ flow continuity in small-calibre corkscrew-like collaterals. A monochrome image better delineates the corkscrew shape of the lumen with superior resolution [22]. It seems that cSMI is used mostly in comparing SMI with PD or other tools because in parallel it depicts the same vessels as a colour overlay image. The value of mSMI for the assessment of synovial vascularity is not discussed but helps to catch the eye and specify flow signals from small to large vessels in the grey map. The diagnostic use of mSMI in detecting inflammatory vascular lesions in vasculitis should be evaluated. Recent studies demonstrated the use of SMI in detecting vascularization of the dermis whereas conventional Doppler modalities cannot [52,53]. Corvino et al. evaluated the dermis using the VI value at the level of five body areas—forehead, forearm, palm, buttock, and thigh—and made an assumption that SMI is a sensitive technique in quantifying disease activity [53]. There is a group of diseases in rheumatology with involvement of the derma, especially systemic sclerosis (SSc). Studies are strongly encouraging and suggest that the US could potentially be more sensitive than traditionally used clinical assessment alone, especially the modified Rodnan skin score [54]. The added value of dermis vascularization changes using high-resolution ultrasound could improve the use of US modalities in early SSc management. The monochrome mode of SMI better visualizes small vascular dots in the background of lower echogenicity tissue—it is valuable for skin assessment. 

The quality of the image depends on the model, transducer, and settings, but mainly the skills of the operator to combine everything into a whole. SMI is available on the Canon Aplio Platinum and i-series models. Reaching the extremely high spatial resolution also depends on using the appropriate transducer. The ultra- or multi-high transducers (frequencies of 20 MHz and greater) offer unprecedented spatial resolution and enhanced sensitivity for new advanced Doppler technologies such as SMI [55,56]. Several authors should have mentioned the machine model [17] and transducer [17,22,23]. A significant proportion of studies did not present their US technique and settings, which creates a great disadvantage: 61% of reports mentioned alignment for SMI assessment and 50% showed settings. In addition, not all of the studies reported the scanned planes, which makes the comparison of the results not completely accurate. An insufficient methodology diminishes reproducibility and research integrity, which are milestones of every scientific study [57]. New discoveries demand detailed methodologies and homogeneity among studies for the learning and update of the research in the most correct way.

However, several limitations could have biased our study. A meta-analysis was not performed because of the heterogeneity between studies in their comparable sample size, disease activity, and tools. We also highlight the differences in technical and/or methodological data in the analysed studies. No study has examined the efficacy of treatment, for example, the real time of sustained remission. Future studies should compare the use of SMI in monitoring treatment efficacies through long-term cohort studies. Only studies written in English were included, and the other research in other languages might have been overlooked. An extensive search of conference abstracts was not conducted.

## 5. Conclusions

This is the first systematic review designed to evaluate the use of SMI in assessing rheumatic diseases. Analysed studies have shown the potential of SMI for diagnosing inflammatory arthritis (exclusively rheumatoid arthritis) in its early phases and evaluating true remission. There is no reason not to believe that in the near future, SMI will become more widely available and new fields of rheumatology (Sjogren’s disease, or even vasculitis) will be discovered. There are many new unexplored areas in rheumatology for possible use advantages of SMI, including SpA, and connective tissue diseases. Further investigations with clearly defined characteristics of larger samples, and follow-up during treatment are needed. 

## Data Availability

Not applicable.

## Supplementary Materials

The following supporting information can be downloaded at: https://www.mdpi.com/article/10.3390/medicina59091641/s1, Table S1: Baseline characteristics of identified studies and the US technique.
