# Supplementary material for: The Use of Superb Microvascular Imaging in Evaluating Rheumatic Diseases: A Systematic Review
Source: Medicina (Kaunas). 2023 Sep 11;59(9):1641. doi: 10.3390/medicina59091641 (PMC10537482; doi:10.3390/medicina59091641)
Supplement: Supplementary file 1 [file medicina-59-01641-s001.zip › medicina-2568402-supplementary.pdf]

Supplemental Table S1 Baseline characteristics of identified studies and the US technique

Author<br>Country, year	Disease	Disease course/<br>±Controls<br>Sample size	Model	Transducer, MHz	Scanned joint or region	Planes	SMI modes	Comparing instrumental tools	SMI scoring system
**Matsuo et al. (11)**<br>Japan, 2022	RA	Relapse<br>210	Aplio 500	12 MHz linear (18L7)	Bilaterally: MCP II-V, radial wrist, ulnar wrist, MTP II-V, Lisfranc, cuneonavicular, Chopart, and ankle joints	N/A	N/A	Only US SMI examination (for comparing machine learning classifiers)	Semi-quantitive scale (four-point 0-3)
**Diao et al. (16)**<br>China, 2022	RA	Active/<br>Remission<br>63/48	Aplio i700	Multifrequency 12-14 MHz linear	Bilaterally: MCP I-V, PIP I-V, knee, wrist, and ankle joints	Axial, longitudinal	N/A	SMI vs. CEUS	Semi-quantitive scale (four-point 0-3)
**Matsuo et al. (12)**<br>Japan, 2021	RA	Remission/<br>Relapse<br>112/9	Aplio 500	12 MHz linear (18L7)	Bilaterally: MCP II-V, radial wrist, ulnar wrist, MTP II-V, Lisfranc, cuneonavicular, Chopart, and ankle joints	Based on OMERACT	Color	SMI result among groups	Semi-quantitive scale (four-point 0-3)
**Kandemirli et al. (21)**<br>Turkey, 2021	JIA	Active<br>30	Aplio 500	14 MHz	Knee, hip, ankle, wrist, elbow joints; flexor hallucis longus/tibialis posterior tendons	Longitudinal, transverse	N/A	SMI vs. PD	Semi-quantitative scale (four-point 0-3)
**Nas et al. (22)**<br>Turkey, 2021	BD	Active<br>14	Aplio 500 or Aplio i600	High-frequency linear	Distal posterior tibial, anterior tibial, distal superficial femoral, and peroneal arteries	N/A	Both	SMI vs. PD vs. DSA	Semi-quantitative scale (types I-IV)
**Wang et al. (17)**<br>China, 2021	AS	Remission/<br>Controls<br>60/30	N/A	N/A	Sacroiliac joint	N/A	N/A	SMI vs. SWE result among groups	-
**Ünal et al. (23)**<br>Turkey, 2020	JIA	N/A – Swelling or Arthralgia/<br>Controls<br>22/24	Aplio 500	N/A	Knee joint; infraarticular and supraarticular soft tissue (fat pads)	Longitudinal, transverse	N/A	SMI vs. PD vs. SWE	Vascularity index
**Jin X al. (18)**<br>China, 2020	RA	Active/<br>Controls<br>41/20	Aplio 500	14 MHz high-frequency	Suprapatellar recess of the knee joints	N/A	N/A	SMI vs. PD vs. CD	Semi-quantitive scale (four-point, 0-III grades)
**Oo et al. (25)**<br>Australia, 2020	OA	N/A<br>89	Aplio 500	Multi-frequency linear (using 10 MHz with 14 L5 MHz transducer)	Suprapatellar, medial and lateral parapatellar knee recesses	Longitudinal, transverse	N/A	SMI vs. PD vs. radiography and MRI	Semi-quantitive scale (four-point 0-3)

**Matsuo et al. (13)** Japan, 2020	RA	N/A 293	Aplio 500	12 MHz linear	Bilaterally: MCP II-V, radial wrist, ulnar wrist, MTP II-V, Lisfranc, cuneonavicular, Chopart, and ankle joints	N/A	Color	SMI with B-mode among groups	Semi-quantitive scale (four-point 0-3)
**Ustabaşıoğlu et al. (24)** Turkey, 2020	pSS	N/A/ Controls 20/20	Aplio 500	5-14 MHz linear	Parotid and submandibular glands	Longitudinal, transverse	N/A	SMI vs. PD vs. CD	Vascularity index
**Lee et al. (27)** South Korea, 2019	RA	N/A/ Controls 56/5	Aplio 500	14 MHz	Bilaterally: wrists, MCP, and PIP	N/A	N/A	SMI vs. PD vs. B-mode	Semi-quantitive scale (four-point 0-3)
**Horie et al. (14)** Japan, 2019	RA	Prodrome of arthritis 15 rats	Aplio i800	Ultrahigh - frequency (8-24 MHz, used 12 MHz)	Ankle joints	Longitudinal	N/A	SMI vs. PD	Vascularity index
**Yu et al. (19)** China, 2018	RA	Remission 26	Aplio 500	5-14 MHz broadband linear	Bilaterally: wrists, MCP, and PIP	N/A	N/A	SMI vs. PD	Semi-quantitive scale (four-point 0-3)
**Lim et al. (28)** United Kingdom, 2018	RA OA PsA IA Only arthralgia	N/A - Arthralgia/ Controls 83/10	Aplio 500	18 MHZ	Radiocarpal, MCP, PIP, and CMC joints; In 5 patients – tarso-metatarsal, talo-navicular or tibio-talar joints In 2 patients – acromioclavicular joints	Longitudinal, transverse	N/A	SMI vs. PD	Semi-quantitive scale (four-point 0-3)
**Yokota et al. (15)** Japan, 2018	RA and Non-RA*	Active RA/Non-RA 27/12	Aplio 300	9 or 18 MHz linear	Bilaterally: MCP, PIP, IP joints, wrists, elbows, knees	Longitudinal	Color	SMI vs. PD	Semi-quantitative scale (four-point 0-3)
**Orlandi et al. (26)** Italy, 2017	RA	N/A/ Controls 30 RA (15 early RA, 15 RA under RTX treatment)/5	Aplio 400	7-18 MHz broadband linear-array	Bilaterally: ulnar recess, MCP I-V and PIP I-V joints	Axial, longitudinal	N/A	SMI vs. PD vs. B-mode	Semi-quantitative scale (four-point 0-3)
**Li et al. (20)** China, 2016	RA	N/A/ Controls 75/10	Aplio 500	18 MHz broad band linear array	MCP, PIP joints	Longitudinal	N/A	SMI vs. PD	Semi-quantitative scale (four-point 0-3)

AOSD = adult-onset Still's disease; AS = ankylosing spondylitis; BD = Buerger's disease; CD = color Doppler; CEUS = contrast-enhanced ultrasound; CMC = carpometacarpal; CRP = C-reactive protein; DSA = digital subtraction angiography; ESR = erythrocyte sedimentation rate; GPA = granulomatosis with polyangiitis; IA = inflammatory arthritis; IP = interphalangeal; JIA = juvenile idiopathic arthritis; MCP = metacarpophalangeal; MCTD = mixed connective tissue disease; MRI = magnetic

resonance imaging; MTP = metatarsophalangeal; N/A = not applicable; OA = osteoarthritis; PD = power Doppler; PIP = proximal interphalangeal; PR = polymyalgia rheumatica; PsA = psoriatic arthritis; pSS = primary Sjogren syndrome; RA = rheumatoid arthritis; RTX = rituximab; SLE = systemic lupus erythematosus; SMI = superb microvascular imaging; SpA = spondyloarthropathy; SWE = shear-wave elastography; UA = unclassified arthritis; US = ultrasound.

*2 SpA, 2 PR, 2 OA, 1 SLE, 1 MCTD, 1 AOSD, 1 GPA, 1 UA, 1 sarcoidosis.
